# Research on Deep Learning Automatic Vehicle Recognition Algorithm Based on RES-YOLO Model

**DOI:** 10.3390/s22103783

**Published:** 2022-05-16

**Authors:** Yanyi Li, Jian Wang, Jin Huang, Yuping Li

**Affiliations:** 1 College of Surveying and Geo-Informatics, Tongji Univesity, Shanghai 200092, China; 2131939@tongji.edu.cn (Y.L.); 2131946@tongji.edu.cn (Y.L.); 2 College of Geodesy and Geomatics, Shandong University of Science and Technology, Qingdao 266590, China; 3 Ocean College, Zhejiang University, Zhoushan 316021, China; ethan.j.huang@foxmail.com

**Keywords:** deep learning, automatic driving, target recognition, adaptive loss function, YOLO, vehicle detection model

## Abstract

With the introduction of concepts such as ubiquitous mapping, mapping-related technologies are gradually applied in autonomous driving and target recognition. There are many problems in vision measurement and remote sensing, such as difficulty in automatic vehicle discrimination, high missing rates under multiple vehicle targets, and sensitivity to the external environment. This paper proposes an improved RES-YOLO detection algorithm to solve these problems and applies it to the automatic detection of vehicle targets. Specifically, this paper improves the detection effect of the traditional YOLO algorithm by selecting optimized feature networks and constructing adaptive loss functions. The BDD100K data set was used for training and verification. Additionally, the optimized YOLO deep learning vehicle detection model is obtained and compared with recent advanced target recognition algorithms. Experimental results show that the proposed algorithm can automatically identify multiple vehicle targets effectively and can significantly reduce missing and false rates, with the local optimal accuracy of up to 95% and the average accuracy above 86% under large data volume detection. The average accuracy of our algorithm is higher than all five other algorithms including the latest SSD and Faster-RCNN. In average accuracy, the RES-YOLO algorithm for small data volume and large data volume is 1.0% and 1.7% higher than the original YOLO. In addition, the training time is shortened by 7.3% compared with the original algorithm. The network is then tested with five types of local measured vehicle data sets and shows satisfactory recognition accuracy under different interference backgrounds. In short, the method in this paper can complete the task of vehicle target detection under different environmental interferences.

## 1. Introduction

In the 1960s, researchers began to conduct preliminary research on vehicle target detection [1,2]. It is a wide consensus that vehicle target detection is the basis of high-end technologies such as traffic big data platform construction, vehicle information management, and automatic driving [3,4]. Automatic vehicle target recognition is highly difficult because of errors, such as vehicle movement error, ambient light error, and camera field angle change error. Existing recognition methods are hardly able to distinguish vehicle position in real scenes. It also requires manual means for visual interpretation to distinguish vehicles in complex scenes. Therefore, combining images with the deep learning method for automatic vehicle discrimination is feasible. The convolutional neural network (CNN) is mainly used to perform target-recognition tasks in real vehicle scenes.

Mainstream vehicle target detection methods mainly use object features for vehicle target recognition. These methods fall under two categories: the first is to collect a certain number of feature samples based on traditional manual extraction and then classify them with a traditional classifier. The traditional classifier mainly quantifies and manually calibrates the edge features [5,6], light and shadow features [7,8], and size features [9,10] of vehicles, and builds a feature operator, such as a wavelet operator, a HOG (histogram of oriented gradient) operator [11], an LBP (local binary patterns) operator [12], and a SIFT (scale-invariant feature transform) operator [13], and then uses this feature operator to recognize the vehicle target. The second category is based on deep learning. It has two types. One is the double detection method represented by CNN, and the other is the single detection method that transforms the problem into a regression problem. For the double detection method, the first detection is to extract the identification frame with high confidence of the detected object on the image. Standard methods to extract the identification frame area include SS (selective search) [14], RPN (region proposal network) [15], etc. The second detection is to distinguish again according to the characteristics in the identified area to be determined. At present, representative double detection frameworks mainly include R-CNN (region-based revolutionary networks), SPP-NET (spatial pyramid pooling networks) [16], Fast R-CNN (fast region-based revolutionary networks) [17], Faster R-CNN (faster region-based revolutionary networks) [17], etc. For single detection methods, representative detection frameworks currently include SSD (single shot multi-box detector) [18] and YOLO (you only look once) [19]. The double detection method has some advantages over the single detection method in detection accuracy, but there is a computational bottleneck. Although the accuracy of the single detection method is slightly low, its detection effect is better. As a representative of the single detection network, the YOLO network still has problems, such as a long training time and difficulty in discriminating specific targets in multiple scenes. If the single detection methods such as YOLO can be optimized and the interference of non-target errors in the detection can be effectively suppressed by specific means, the efficiency and accuracy will be improved.

Targeting the problem of automatic vehicle target detection, Chinese authors in recent years have conducted extensive research using the method of deep learning. Fengbin Zhu et al. [20] proposed a Fast R-CNN vehicle detection method, accelerated by spatio-temporal fusion, which achieved the rapid detection of specific targets. Yanmei Jin et al. [21] further optimized the SSD algorithm and proposed MF-SSD to solve the problem of road small target detection, but did not conduct in-depth research on vehicle detection alone. Yuanyuan Zhao et al. [22] proposed a flame target detection framework based on the YOLO v3 network, which has a good effect in target recognition of flame image, but it needs to be studied in other fields. Chang Yu Cao et al. [23] proposed a method of vehicle identification using the YOLO network and systematically realized traffic flow statistics. Liu Ken et al. [24] used an improved tiny-YOLO network to identify vehicles and achieved good results. On this basis, Jun Liu et al. [25] and others investigated real-time vehicle target detection using the YOLO algorithm, but they were limited to vehicle detection using YOLO, and did not consider much about further optimization. Li Qiutan et al. [26] used the YOLO v4 network to accurately identify various targets at busy intersections, proving that the YOLO v4 network has practical value in traffic intersection monitoring. Moreover, based on the improvement of the YOLO-v4-tiny network structure, Wang Guanbo et al. [27,28] proposed the Trident-YOLO and TRC-YOLO target detection models. These improved network models can be used on mobile devices. By upgrading and improving the follow-up YOLO network model, Carrasco, D. P. et al. [29] used the improved network based on the YOLO v5 structure for vehicle recognition, significantly optimizing the tiny vehicle recognition in the image. Wu Tianhao et al. [30] realized the detection of the vehicle targets by improving the lightweight YOLO v5 network. In addition, they also estimated the distance between vehicles. Other authors have also optimized different network models in recent years, hoping to realize the rapid detection of vehicle targets from different angles [31,32,33,34,35,36,37]. However, generally speaking, developing an improved model from the YOLO network in terms of structure and loss function so as to achieve better automatic vehicle detection remains to be studied.

To summarize, this paper uses a RES-YOLO deep learning detection network, carries out network training through the BDDK100 vehicle detection data set, selects recent improved models [20,21,22] and the latest Faster R-CNN model, compares the optimized RES-YOLO algorithm model with those traditional models, and tests its detection accuracy under different conditions of small and large data volumes to verify its advantages and reliability for vehicle detection. Finally, the robustness of this algorithm in a complex background environment is further proved by using the data of real measurements in different environments.

The method proposed in this paper aims to accurately recognize vehicles in images, which is a fundamental problem in driverless and traffic flow detection. In this paper, the hardware system of small vehicle recognition is designed, and the RES-YOLO data processing algorithm is proposed. The system can be mounted on the vehicle platform for data acquisition and analysis. At this time, it is equivalent to the vehicle identification of a driverless system. The system can also be fixed at the intersection for data collection and can play a role in traffic flow statistics. Our proposed method focuses more on solving the problem of accurate vehicle recognition in the driverless system. The method in this paper will serve the same type of fields that need vehicle target recognition. In addition, it will provide them with a vehicle recognition method that considers stability and efficiency. Additionally, our method can also provide a detailed reference for solving the problem of vehicle target recognition based on the vision of tiny mobile devices. For the vehicle recognition problem with more background interference, our method can also better solve it, which contributes to improving the stability of the driverless system.

The structure of the article is as follows. Firstly, the basic principle of the YOLO model is introduced in the first section. Then, the second section presents the RES-YOLO vehicle target detection network proposed in this paper. The third section explains the experimental data set and expounds on this paper’s experimental process. This part compares the processing effect between the algorithm and other methods. In addition, it also shows the results of relevant experiments. Additionally, we use localized multi-scene data to test the RES-YOLO algorithm. Finally, the fourth section gives the critical conclusions of the full text and further discussion.

## 2. Basic Principles of YOLO

The full name of YOLO is You Only Look Once. Its essence is to convert an object detection problem into a regression problem and directly obtain the location of the detection boundary and the detection probability from the image. Firstly, n is defined as the scale factor of the feature layer in the detection process, namely, the feature scale factor. Secondly, W and H are defined as the width and height of the image, and S1 = w/N, S2 = H/n. The YOLO algorithm first divides the image data into S1 * S2 detection grids, and each detection network is set with multiple target anchor frames.

When detecting each target anchor frame, if the center of the object being detected falls into the grid, the surrounding grids will be formed into a grid unit, the boundary of the grid unit will be predicted, and the confidence score will be calculated. The value of these scores represents the accuracy of the object detected in the grid unit. The definition of confidence of the detected object is shown in Formula (1):

(1) $${\delta =\mathrm{Pr}(\mathrm{Object})\times \mathrm{IOU}}_{\mathrm{pred}}^{\mathrm{truth}}$$

where Pr(Object) represents the probability of the existence of the target object in the grid unit and IOU represents the coincidence rate between the frame mark in the model prediction and the actual object.

**Figure 1:**
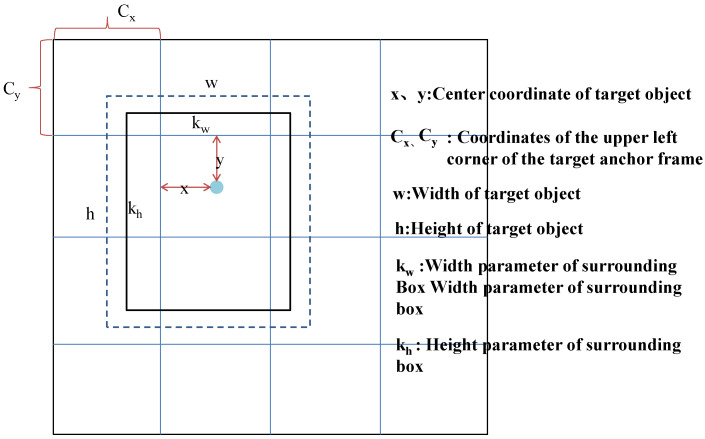
The object position.

Each target anchor box is determined by five main parameters: x,y,w,h,δ, where (x,y) represents the central coordinate of the target object, (w,h) represents the width and height of the target object, and the confidence δ represents the IOU between the prediction frame and any ground real frame. The final detected object boundary frame mark is obtained by the following Formula (2):

(2) $$\{\begin{array}{l} k_{x}{=x+c}_{x}\\ k_{y}{=y+c}_{y}\\ k_{w}{=w\times e}^{w}\\ k_{h}{=h\times e}^{h} \end{array}$$

where $k_{x},{\;k}_{y},\;k_{w},\;k_{h}$ represent the corresponding bounding box parameters and c_x_, c_y_ represent the coordinates at the upper left corner of the target anchor box, as shown in Figure 1. The actual pixel coordinates of the object to be detected in the bounding box can be obtained by multiplying the object bounding box parameter and the feature scale factor n.

After the above process, each target anchor frame can output N parameters ${\mathrm{tn}}_{i},\;i\in \left[1,\;N\right]$, and the occurrence probability of these N types of target objects can be calculated with the normalized exponential function. The formula is shown in Formula (3).

(3) $${\mathrm{Pr}(\mathrm{Class}}_{i}|\mathrm{object})=\frac{e^{{\mathrm{tn}}_{i}}}{\sum_{i=1}^{N}e^{{\mathrm{tn}}_{i}}}$$

After obtaining the corresponding data, index I is calculated according to Formula (4) and sorted. The unqualified target anchor frame is filtered and removed by setting the threshold. The final target anchor frame is the target object to be judged.

(4) $${I=\mathrm{Pr}(\mathrm{Class}}_{i}|\mathrm{object})\times \mathrm{Pr}(\mathrm{object})\times \mathrm{IOU}$$

**Figure 2:**
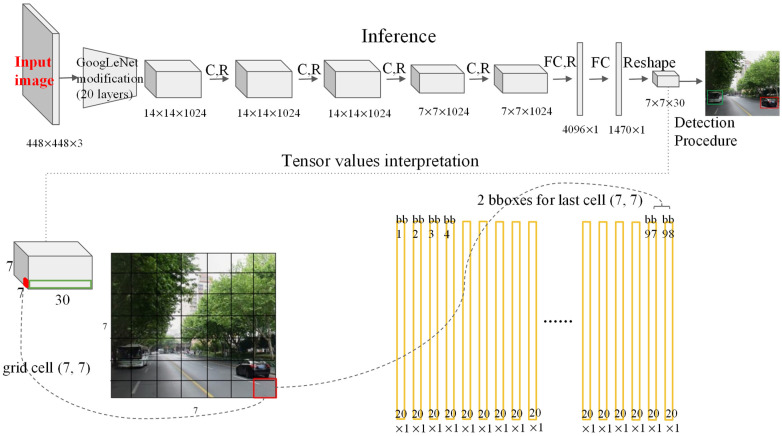
YOLO network structure.

The GoogleNet model for image classification inspires the YOLO network architecture in the structure. The network has 24 convolution layers and finally two fully connected layers. The network does not use the initial module of GoogleNet, but uses 1 × 1 reduction layers and 3 × 3 convolution layers. The complete network structure flow is shown in Figure 2.

## 3. Optimizing RES-YOLO Deep Learning Network for Multi-Vehicle Target Detection

### 3.1. Basic Structure of RES-YOLO Network

The main reasons for choosing the YOLO algorithm are as follows:

(1) In dealing with the regression problem, there is no need to execute complex processes, and the results can be obtained quickly by running directly on the image to be detected;
(2) YOLO algorithm detection is based on the whole image instead of a sliding window, and carries out feature coding on the target and surrounding neighborhood information to improve the detection accuracy;
(3) YOLO algorithm has a low decomposition ability and strong robustness for non-detection category data.

The research mainly optimizes the feature extraction and detection network in the YOLO network, and selects ResNet50 to replace the traditional YOLO network for feature extraction in the target recognition process. *Activation_ 40_ relu* is used as the feature extraction layer to output the image data after 16 instances of down-sampling to build a new RES-YOLO network. Some feature layers in YOLO are still used in the detection network. This ensures the excellent performance of the feature extraction network and retains the advantages of the YOLO network. At the same time, the ResNet50 feature extraction network selected in this paper is consistent with the traditional network structure. The specific reasons for choosing the ResNet50 network for improvement will be discussed later in Section 3.2.

**Figure 3:**
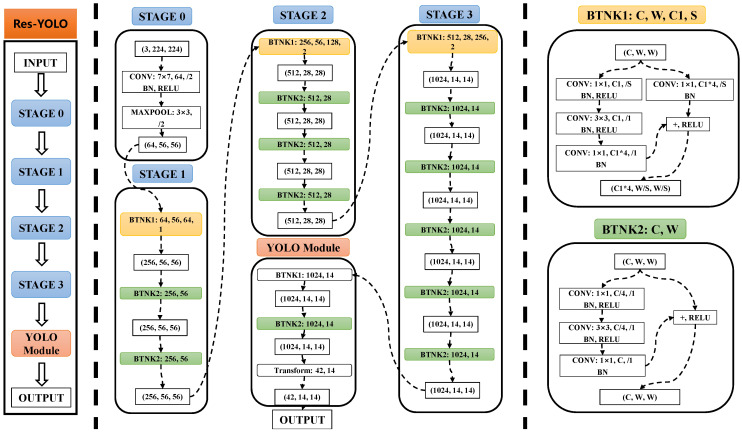
Schematic diagram of RES-YOLO network structure.

The improved network structure is shown in Figure 3. The RES-YOLO network comprises five parts, including stage 0 layer, stage 1 layer, stage 2 layer, stage 3 layer, and stage 4 layer. After data input, the image of each frame is processed through a multi-layer convolution operation. The operation sequence of each layer is as follows:

(1) Stage 0 layer: convolution process for 1 time and maximum pooling process for 1 time;
(2) Stage 1 layer: BTNK1 process for 1 time and BTNK2 process for 2 times;
(3) Stage 2 layer: BTNK1 process for 1 time and BTNK2 process for 3 times;
(4) Stage 3 layer: BTNK1 process for 1 time and BTNK2 process for 5 times;
(5) Stage 4 layer: BTNK1 process for 1 time and BTNK2 process for 2 times, and finally processed by YOLO module.

As shown in the rightmost sub-graph of Figure 3, the complex structure of the original ResNet type network is intensively designed in the network design, and the central operation part of the network is designed into two operation units, BTNK1 and BTNK2. After reading the data, BTNK1 first performs two-way convolution operations, one of which continues to convolute twice and then turns into the activation function ReLU. The other turns directly into the activation function ReLU and outputs the results after being processed by the activation function. After reading the data, the BTNK2 unit first performs two operations, one of which is convoluted three times and then transferred to the activation function ReLU. The other is directly transferred to the activation function ReLU. The result is output after being processed by the activation function. When processing, the two operation units will call a particular memory buffer to effectively carry out the parallel operation to improve operation and processing efficiency. It is also convenient to carry out unit testing and quickly locate the fault node during debugging.

Because the vehicle detection part of the YOLO module is generally a tiny CNN, and its complexity is much lower than that of the feature extraction network, this paper uses some convolution layers and YOLO v2 unique layers for optimization. Finally, the RES-YOLO network structure suitable for performing the vehicle detection task is designed, as shown in Appendix A. After optimization, the number of network layers is slightly reduced, and the performance is improved to a certain extent. A total of 150 layers are involved. To provide a more efficient and robust detection network for vehicle detection, YOLO v3\v4\v5 and other networks with complex network structures are not used. Subsequent experiments have confirmed that, with our optimization, there is no need to use a more complex YOLO network structure for detection. This part of the discussion and experiment will be explained later.

In this part, the details of the RES-YOLO network structure proposed in this paper can be seen in Appendix A. The local structures of BTNK1 and BTNK2 proposed here, as the characteristic structures of the RES-YOLO network, are our method’s characteristics. At the same time, the STAGE0 ~ 4 layers in the RES-YOLO network processing are also the characteristics of our method. Finally, the YOLO module is consistent with the traditional YOLO v3\v4\v5 and other network structures. Here, only a part is spliced for use.

### 3.2. Data Enhancement

**Figure 4:**
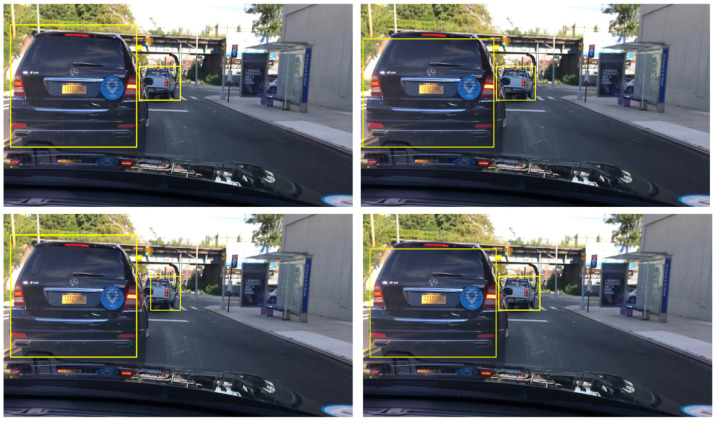
Data enhancement.

This paper enhances the data used in training. In the training process, the random transformation of the original training data is used to improve the data size of the training set to improve the effect of network training. The training data enhancement of the algorithm is mainly carried out by randomly turning the image horizontally and its corresponding frame label information, and expanding the training data set as much as possible while ensuring the information entropy of the original data. The enhancement effect is shown in Figure 4.

### 3.3. Constructing Adaptive Loss Function

In the deep learning network of the YOLO series, the loss function is composed of four variables ${\mathrm{loss}}_{\mathrm{xy}}{,\;\mathrm{loss}}_{\mathrm{wh}}{,\;\mathrm{loss}}_{\mathrm{conf}}{,\;\mathrm{loss}}_{\mathrm{class}}$. They correspond to four loss function terms, and the corresponding influencing factors are: the coordinates of the center position of the detection frame mark, the size of the detection frame mark, the confidence, and the type of the detection object. The constructed loss function is shown in Formula (5):

(5) $$\begin{array}{l} {\mathrm{Loss}=\mathrm{loss}}_{\mathrm{xy}}{+\mathrm{loss}}_{\mathrm{wh}}{+\mathrm{loss}}_{\mathrm{conf}}{+\mathrm{loss}}_{\mathrm{class}}\\ =\frac{1}{2}\cdot \frac{1}{N_{T}}\cdot \lambda \overset{S^{2}}{\underset{\mathrm{coord}}{\sum }}\overset{B}{\underset{j=0}{\sum }}\overset{\mathrm{obj}}{\underset{\mathrm{ij}}{\prod }}\left[{\left(x_{\mathrm{ij}}-{\tilde{x}}_{\mathrm{ij}}\right)}^{2}+{\left(y_{\mathrm{ij}}-{\tilde{y}}_{\mathrm{ij}}\right)}^{2}\right]\\ +\frac{1}{2}\cdot \frac{1}{N_{T}}\cdot \lambda \overset{S^{2}}{\underset{i=0}{\sum }}\overset{B}{\underset{j=0}{\sum }}\overset{\mathrm{obj}}{\underset{\mathrm{ij}}{\prod }}\left[{\left(w_{\mathrm{ij}}-{\tilde{w}}_{\mathrm{ij}}\right)}^{2}+{\left(h_{\mathrm{ij}}-{\tilde{h}}_{\mathrm{ij}}\right)}^{2}\right]\\ +\frac{1}{2}\cdot \frac{1}{N_{T}{+N}_{\mathrm{FT}}}\cdot \left[\lambda \overset{S^{2}}{\underset{\mathrm{obj}}{\sum }}\overset{B}{\underset{j=0}{\sum }}\overset{\mathrm{obj}}{\underset{\mathrm{ij}}{\prod }}{\left(C_{\mathrm{ij}}-{\tilde{C}}_{\mathrm{ij}}\right)}^{2}+\lambda \overset{S^{2}}{\underset{\mathrm{noobj}}{\sum }}\overset{B}{\underset{j=0}{\sum }}\overset{\mathrm{noobj}}{\underset{\mathrm{ij}}{\prod }}{\left(C_{\mathrm{ij}}-{\tilde{C}}_{\mathrm{ij}}\right)}^{2}\right]\\ +\frac{1}{2}\cdot \frac{1}{N_{T}}\cdot \lambda \overset{a^{2}}{\underset{\mathrm{class}}{\sum }}\overset{B}{\underset{j=0}{\sum }}\overset{\mathrm{obj}}{\underset{\mathrm{ij}}{\prod }}{{(P}_{\mathrm{ij}}\left(c)+{\tilde{P}}_{\mathrm{ij}}(c)\right)}^{2} \end{array}$$

where $N_{T}{,\;N}_{F}\;$ represent the number of positive samples and the number of negative samples, $S^{2}{=S}_{w}\times S_{h}\;$, ${\mathrm{loss}}_{\mathrm{xy}}{,\;\mathrm{loss}}_{\mathrm{wh}}{,\;\mathrm{loss}}_{\mathrm{conf}}{,\;\mathrm{loss}}_{\mathrm{class}}$ represent the weight coefficient corresponding to the loss function item under the influence of the central position coordinate of the detection frame mark, the size and confidence of the detection frame mark, and the type of the detection object, i represents the corresponding grid number and j represents the corresponding detection unit number in the grid i. When there is a positive sample in the detection unit, $\overset{\mathrm{obj}}{\underset{\mathrm{ij}}{\prod }}$ equals 1; otherwise, it equals 0; when there is a negative sample in the detection unit, $\overset{\mathrm{noobj}}{\underset{\mathrm{ij}}{\prod }}$ equals 1; otherwise, it equals 0.

The essence of the YOLO algorithm is to grid the whole image data for one detection, so that the number of positive samples in the detection unit is 0 or 1, while the number of negative samples is much greater than 1. This results in remarkable differences between the number of positive and negative samples in the whole image data, with an order of magnitude of difference of up to $10^{2}{\sim 10}^{3}$. The imbalance between positive and negative samples will reduce the efficiency of the algorithm model training. At the same time, a large number of negative samples will cover up the positive samples, resulting in model degradation after training. In order to solve this problem, the detection network of the traditional YOLO and its subsequent versions (such as YOLO v2, YOLO v3, YOLO v4, etc.) try to improve learning efficiency by controlling two weight proportion coefficients $\lambda_{\mathrm{obj}}{,\;\lambda }_{\mathrm{noobj}}$ to suppress the influence of negative samples on the loss function and increase the contribution of positive samples to the loss function. However, the simple assignment of these two scale coefficients can only be controlled within a certain threshold; otherwise, it will cause overfitting or training errors. For this reason, this paper introduces an adaptive proportional coefficient to adjust $\lambda_{\mathrm{obj}},\;\lambda_{\mathrm{noobj}}$, so as to control the order of magnitude difference between the positive and negative samples.

Firstly, based on Formula (6), it is found that the confidence loss ${\mathrm{loss}}_{\mathrm{conf}}$ is the core to be optimized, which includes both coefficients $\lambda_{\mathrm{obj}},\;\lambda_{\mathrm{noobj}}$, as shown in Formula (7).

(6) $${\mathrm{Loss}}_{\mathrm{conf}}=\frac{1}{2}\cdot \frac{1}{N_{T}{+N}_{\mathrm{FT}}}\cdot \left[\lambda \overset{S^{2}}{\underset{\mathrm{obj}}{\sum }}\overset{B}{\underset{j=0}{\sum }}\overset{\mathrm{obj}}{\underset{\mathrm{ij}}{\prod }}{\left(C_{\mathrm{ij}}-{\tilde{C}}_{\mathrm{ij}}\right)}^{2}+\lambda \overset{S^{2}}{\underset{\mathrm{noobj}}{\sum }}\overset{B}{\underset{j=0}{\sum }}\overset{\mathrm{noobj}}{\underset{\mathrm{ij}}{\prod }}{\left(C_{\mathrm{ij}}-{\tilde{C}}_{\mathrm{ij}}\right)}^{2}\right]$$

Deriving the above formula, we produce:

(7) $$\frac{\partial {\mathrm{Loss}}_{\mathrm{conf}}}{\partial C_{i}}=\frac{1}{N_{T}{+N}_{\mathrm{FT}}}\cdot \left[\lambda \overset{S^{2}}{\underset{\mathrm{obj}}{\sum }}\overset{B}{\underset{j=0}{\sum }}\overset{\mathrm{obj}}{\underset{\mathrm{ij}}{\prod }}{\left(C_{\mathrm{ij}}-{\tilde{C}}_{\mathrm{ij}}\right)}^{2}+\lambda \overset{S^{2}}{\underset{\mathrm{noobj}}{\sum }}\overset{B}{\underset{j=0}{\sum }}\overset{\mathrm{noobj}}{\underset{\mathrm{ij}}{\prod }}{\left(C_{\mathrm{ij}}-{\tilde{C}}_{\mathrm{ij}}\right)}^{2}\right]$$

Through the above formula, it is found that the derivative of confidence is positively correlated with the difference between the predicted value and the true value, so:

(8) $$\Delta W=-\eta_{W}\cdot \frac{\partial {\mathrm{Loss}}_{\mathrm{conf}}}{\partial W}=-\eta_{W}\cdot \frac{\partial {\mathrm{Loss}}_{\mathrm{conf}}}{\partial C_{\mathrm{ij}}}\cdot \frac{\partial C_{\mathrm{ij}}}{\partial W}$$

where ΔW represents the increment of the loss function to the variable and $\eta_{W}$ represents the learning rate. According to the above three formulas, if the difference between the number of positive and negative samples is too significant, the gradient characteristics will not be prominent, and finally the detection will fail. Based on this, the adaptive adjustment coefficient shown below is constructed to balance the impact of the difference.

(9) $${\alpha =(|C}_{\mathrm{ij}}-{\tilde{C}}_{\mathrm{ij}}{\left|\right)}^{\gamma^{b}}$$

The adaptive adjustment coefficient can be generated adaptively according to the difference between the predicted value and the actual value. Here, b effectively controls the value of the adjustment coefficient by controlling the value of the exponential part, and α is effectively controlled by using the high gradient change principle of the exponential function. When the sample under test is a positive one, ${\tilde{C}}_{\mathrm{ij}}=1$; when it is a negative one, ${\tilde{C}}_{\mathrm{ij}}=0$. After introducing the new adaptive scale factor, we have:

(10) $$\begin{array}{l} {\alpha =(|C}_{\mathrm{ij}}-{\tilde{C}}_{\mathrm{ij}}{\left|\right)}^{\gamma^{b}}{\mathrm{Loss}}_{\mathrm{conf}}^{*}\\ {=\alpha \text{×}\mathrm{Loss}}_{\mathrm{conf}}\\ =\frac{1}{2}\cdot \frac{1}{N_{T}{+N}_{\mathrm{FT}}}\cdot \left[\lambda \overset{S^{2}}{\underset{\mathrm{obj}}{\sum }}\overset{B}{\underset{j=0}{\sum }}\overset{\mathrm{obj}}{\underset{\mathrm{ij}}{\prod }}{\left(C_{\mathrm{ij}}-1\right)}^{\gamma^{b}+2}+\lambda \overset{S^{2}}{\underset{\mathrm{noobj}}{\sum }}\overset{B}{\underset{j=0}{\sum }}\overset{\mathrm{noobj}}{\underset{\mathrm{ij}}{\prod }}{\left(C_{\mathrm{ij}}-0\right)}^{\gamma^{b}+2}\right] \end{array}$$

Deriving the above formula, we have:

(11) $$\frac{\partial {\mathrm{Loss}}_{\mathrm{conf}}^{*}}{\partial W}=\frac{\gamma^{b}+2}{{2(N}_{T}{+N}_{\mathrm{FT}})}\cdot \left[-\lambda \overset{S^{2}}{\underset{\mathrm{obj}}{\sum }}\overset{B}{\underset{j=0}{\sum }}\overset{\mathrm{obj}}{\underset{\mathrm{ij}}{\prod }}{\left(1-C_{\mathrm{ij}}\right)}^{\gamma^{b}+1}+\lambda \overset{S^{2}}{\underset{\mathrm{noobj}}{\sum }}\overset{B}{\underset{j=0}{\sum }}\overset{\mathrm{noobj}}{\underset{\mathrm{ij}}{\prod }}{\left(C_{\mathrm{ij}}-0\right)}^{\gamma^{b}+1}\right]$$

**Figure 5:**
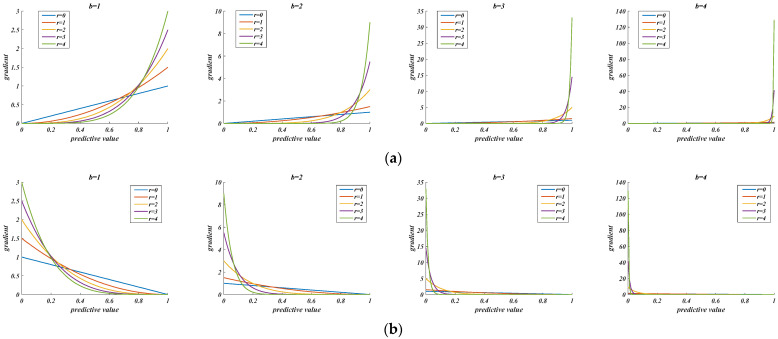
Derivative variation of loss function. (**a**) Positive sample; (**b**) Negative sample.

Therefore, after adding the adaptive scale coefficient, the larger the difference between the predicted and the actual value, the larger the derivative of the loss term, and vice versa. The derivative curve of the loss function is shown in Figure 5.

In Figure 5, it is not difficult to find an excessively large b, which will result in an excessively large positive and negative samples gradient variation, whereas an excessively small b will result in insensitive gradient variation. When γ=0, the loss function is consistent with the traditional function, showing that Formula (10) is essentially a generalized form of the loss function of confidence. Finally, after testing, the best effect is achieved when b = 2, γ=2. The final definition of the loss function in this model is shown in Formula (12).

(12) $$\begin{array}{l} {\mathrm{Loss}=\mathrm{loss}}_{\mathrm{xy}}{+\mathrm{loss}}_{\mathrm{wh}}{+\mathrm{loss}}_{\mathrm{conf}}{+\mathrm{loss}}_{\mathrm{class}}\;\\ =\frac{1}{2}\cdot \frac{1}{N_{T}}\cdot \lambda \overset{S^{2}}{\underset{\mathrm{coord}}{\sum }}\overset{B}{\underset{j=0}{\sum }}\overset{\mathrm{obj}}{\underset{\mathrm{ij}}{\prod }}\left[{\left(x_{\mathrm{ij}}-{\tilde{x}}_{\mathrm{ij}}\right)}^{2}+{\left(y_{\mathrm{ij}}-{\tilde{y}}_{\mathrm{ij}}\right)}^{2}\right]\\ +\frac{1}{2}\cdot \frac{1}{N_{T}}\cdot \lambda \overset{S^{2}}{\underset{i=0}{\sum }}\overset{B}{\underset{j=0}{\sum }}\overset{\mathrm{obj}}{\underset{\mathrm{ij}}{\prod }}\left[{\left(w_{\mathrm{ij}}-{\tilde{w}}_{\mathrm{ij}}\right)}^{2}+{\left(h_{\mathrm{ij}}-{\tilde{h}}_{\mathrm{ij}}\right)}^{2}\right]\\ +\frac{1}{2}\cdot \frac{1}{N_{T}{+N}_{\mathrm{FT}}}\cdot \left[\lambda \overset{S^{2}}{\underset{\mathrm{obj}}{\sum }}\overset{B}{\underset{j=0}{\sum }}\overset{\mathrm{obj}}{\underset{\mathrm{ij}}{\prod }}{\left(C_{\mathrm{ij}}-{\tilde{C}}_{\mathrm{ij}}\right)}^{2^{2}+2}+\lambda \overset{S^{2}}{\underset{\mathrm{noobj}}{\sum }}\overset{B}{\underset{j=0}{\sum }}\overset{\mathrm{noobj}}{\underset{\mathrm{ij}}{\prod }}{\left(C_{\mathrm{ij}}-{\tilde{C}}_{\mathrm{ij}}\right)}^{2^{2}+2}\right]\\ +\frac{1}{2}\cdot \frac{1}{N_{T}}\cdot \lambda \overset{a^{2}}{\underset{\mathrm{class}}{\sum }}\overset{B}{\underset{j=0}{\sum }}\overset{\mathrm{obj}}{\underset{\mathrm{ij}}{\prod }}{{(P}_{\mathrm{ij}}\left(c)+{\tilde{P}}_{\mathrm{ij}}(c)\right)}^{2} \end{array}$$

## 4. Experiment and Results

### 4.1. Experimental Data Set and Sensor

The BDD100K algorithm is used to verify the effectiveness in this paper. Firstly, the data set is divided into two groups: the small sample data are used to pre-verify the algorithm, and large sample data are used to further optimize and evaluate the training model. More details of the data set are shown in Table 1.

First, select some data from the BDD100K data set as small samples for training and testing, so as to pre-verify the feasibility and effectiveness of the algorithm for vehicle detection. Then, take the BDD100K data set as a large sample size data set for model training and verification, so as to verify and compare the general performance of the model algorithm. During the model evaluation, we divide the total data set into a training set, a verification set and a test set, with the ratio of 6:1:3. Table 1 also reflects the evaluation method of train/val split.

**Figure 6:**
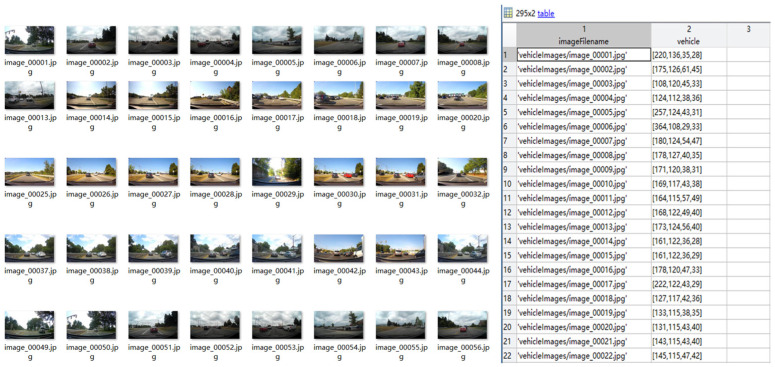
Custom data set in this research.

The data used in the pre-verification are referred to here as small data test. The data set selects the category of small cars through the annotation file “*bdd100k_labels_images_val.json*” of the original data of BDD100K, and reorganizes the customized small batch training and test data set. For the large sample size data set, which is referred to here as large data test, all the data used for vehicle target judgment in the BDD100K data set are directly used. The reconstructed sample data mainly include the path of the picture source and the location information of the vehicle target mark, as shown in Figure 6 below.

**Figure 7:**
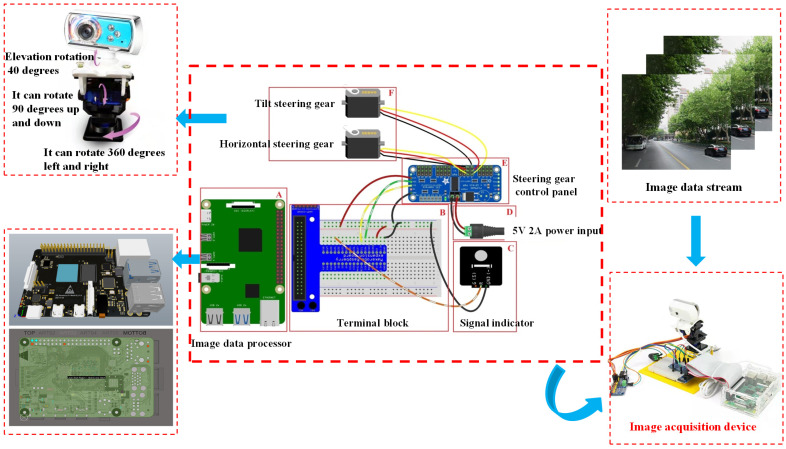
The visual sensor structure used in this paper.

As shown in Figure 7, in this study, the convenient vision sensor, which we built ourselves, is used for image acquisition. The structure of the sensor system is divided into six parts: part A is the image processor, which is mainly used to detect the vehicle target on the collected image. Part B is the terminal block, which is used to connect various sensor parts. Part C is the power indicator, which is used to display the working state of the system. Part D is the power input unit, which needs to input a 5 V 2 A power supply to ensure that the system works normally. Part E is the steering gear control board, which is used to control the movement of the steering gear base with two degrees of freedom. Part F is the steering gear part, which can adjust the viewing angle position of the camera. Using this set of sensor devices, the vehicle image data can be collected for a long time on the premise of low power consumption, so as to prepare the data for subsequent experiments.

### 4.2. Determination of Feature Extraction Network

In order to better evaluate the impact of different extraction network settings on the YOLO model, a lightweight, a robust, and a complex extraction network are selected for testing. Based on the two factors of operation time and operation accuracy, the optimal network structure is selected by comparison. The leading information of the test is shown in Table 2 and Table 3.

Considering the training time, the small sample data group is uniformly selected for training throughout the study, and the trained models are tested respectively. Finally, the optimal network matching is selected for the next experiment.

**Figure 8:**
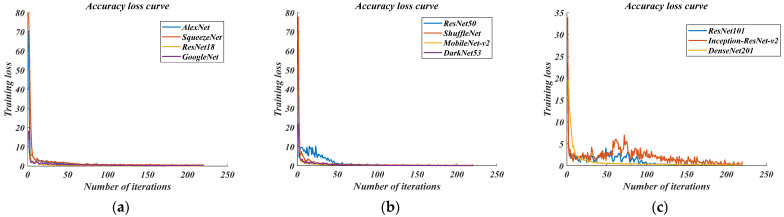
Accuracy–loss curve in this research. (**a**) Lightweight network; (**b**) Robust network; (**c**) Complex network.

Through experiments, the detection–accuracy–loss curve and the accuracy–recall curve of the three groups of network models are obtained, as shown in Figure 8 below.

**Figure 9:**
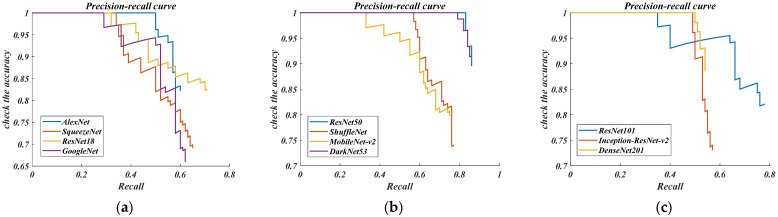
Precision and recall (PR) curve in this research. (**a**) Lightweight network; (**b**) Robust network; (**c**) Complex network.

The accuracy–loss curve, whether for the lightweight network, robust network, or complex network, can converge within 250 iterations, which also shows that the above networks can objectively complete the model’s training. However, we can see from Figure 8c that the complex network cannot complete the convergence of training loss in the first 50 iterations, indicating that there are problems such as over-fitting and inability to fit in the recognition process. Therefore, it is preliminarily determined that the complex network is not considered when dealing with vehicle recognition. At the same time, it can also be found from Figure 8a,b that the lightweight AlexNet and SquuzeNet networks, and the robust ResNet50 and ShuffleNet networks have an extensive convergence range for training loss in the first 50 iterations, which also confirms that these four networks are more suitable for dealing with vehicle recognition problems. In order to further determine the optimal network, the accuracy–recall curve under the same test conditions is compared, as shown in Figure 9 below.

From Figure 9, except for the robust ResNet50 and DarkNet53 networks, which maintain a detection accuracy above 90% at a recall rate greater than 0.8, the detection accuracy of all other networks decreased significantly before the recall rate reaches 0.8, which obviously fails the task goal of the high-precision vehicle target detection. As shown in Figure 9a, when the recall rate of the lightweight networks reaches 0.6, the detection accuracy cannot exceed 85%, and some instances of detection accuracy cannot even exceed 70%. From Figure 9b, virtually the same thing happens to the complex networks. Therefore, in this paper, lightweight and complex networks are not suitable for vehicle detection. Considering that the ResNet50 network shows good performance in the precision–loss curve and the precision–recall curve, we determine that the optimal network is a robust ResNet50 network. The specific test conditions of the above networks are shown in Table 3.

**Figure 10:**
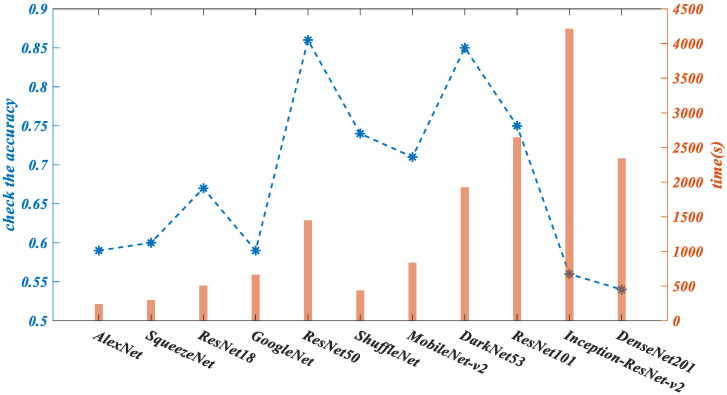
Comparison diagram of preliminary test of small amount of data on network performance.

ResNet50 is a typical residual network structure, in which the data output of a particular layer of the first several layers is directly introduced across multiple layers into the input part of the later data layer, which means that a previous layer will linearly contribute part of the content of the later feature layer. This is performed to solve the problem that the efficiency and detection accuracy decrease with the deepening of the learning complexity. After testing, as shown in Figure 10, the ResNet50 network, as a robust network, has moderate structural complexity and the highest accuracy among the other networks. By integrating such a network as YOLO v2 for modification, we can further optimize the detection effect for particular scenes, such as vehicle target detection, while ensuring efficiency. Finally, the ResNet50 network, with reasonable training time and the highest accuracy, is selected as the feature extraction network to be optimized by the YOLO algorithm. On this basis, an improved new network RES-YOLO is proposed for a comprehensive vehicle detection experiment.

### 4.3. Comprehensive Experimental Process

**Figure 11:**
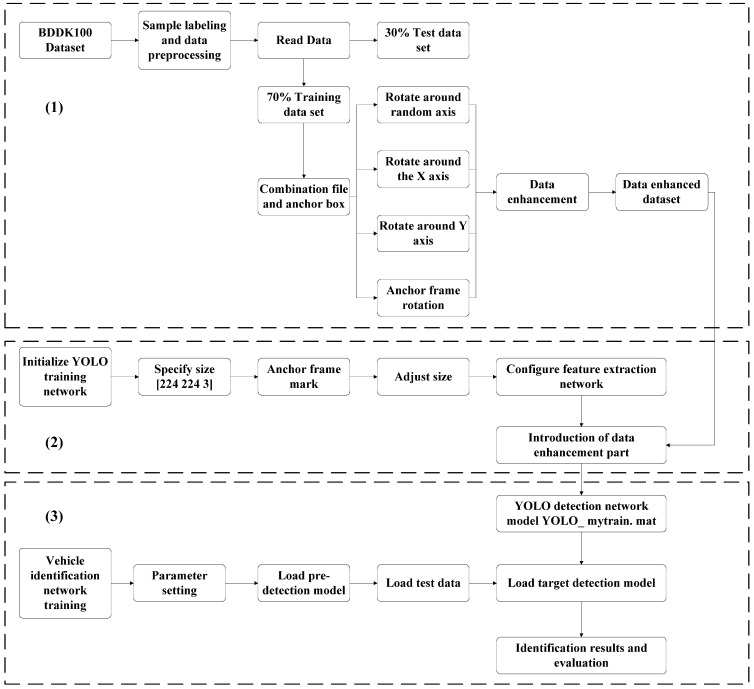
The method flow chart of this paper. The data processing flow includes three parts: (1) data set preprocessing and data enhancement, (2) initialization of the YOLO training network, and (3) vehicle identification network training and testing.

The workflow of the optimized RES-YOLO automatic recognition method is shown in Figure 11. This method can detect vehicle targets for various image data under a variety of different environmental conditions.

Part (1) mainly deals with data sets. After the data are read, they are grouped into a training data set, a test data set, and a verification data set, at a ratio of 6:3:1. Then, the anchor frame of the training data set is calibrated. After calibration, the training data set and its calibration are enhanced from three angles to prepare for subsequent model training.

Part (2) mainly creates the network structure for the RES-YOLO deep learning model. To interface the data sets effectively, the data size input by the designated YOLO framework [224 224 3] is directly used, the anchor box mark of the network is initialized, and the original data set is resized before it is inputted into the model. The ResNet50 network is introduced to extract the data features. After extracting the feature matrix parameters, the data set is trained to obtain the vehicle target automatic recognition model based on deep learning and prepare for subsequent testing and task execution.

Part (3) focuses on vehicle target recognition and model testing. First, the model detected by the ResNet50 network and the target recognition model obtained in the second part are preloaded to detect the test set data. Finally, the results of the labeled samples are compared to obtain the accuracy–loss, test curves, and other data.

### 4.4. Comprehensive Comparative Experiment

**Figure 12:**
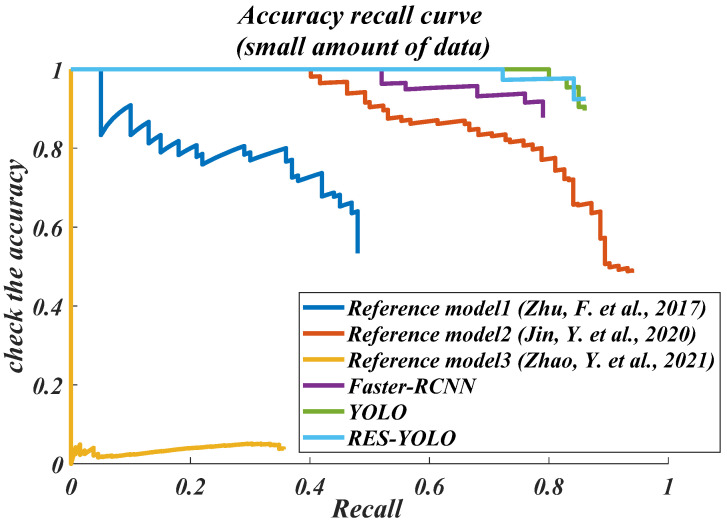
Precision and recall (PR) curve in small data set (small amount of data) [20,21,22].

**Figure 13:**
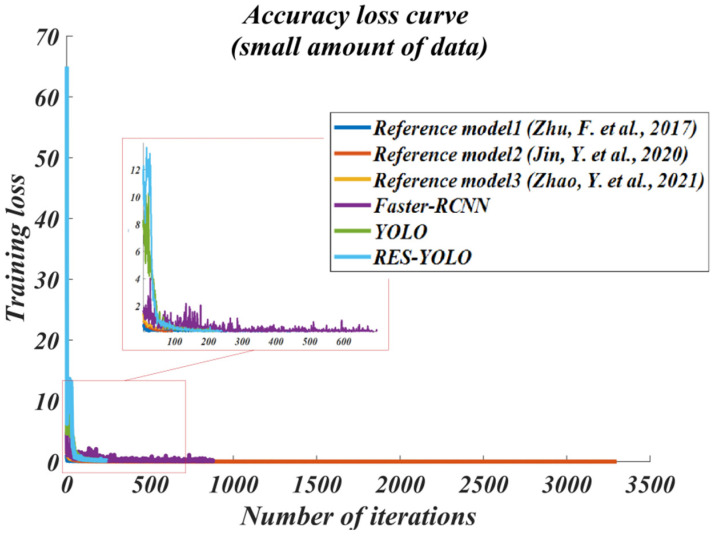
Accuracy–loss curve in small data set (small amount of data) [20,21,22].

**Figure 14:**
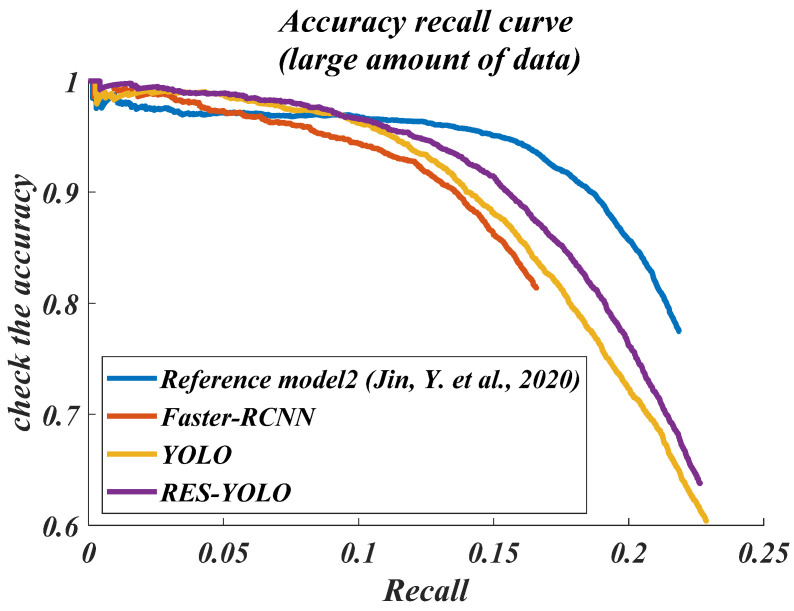
Precision and recall (PR) curve in small data set (large amount of data) [21].

**Figure 15:**
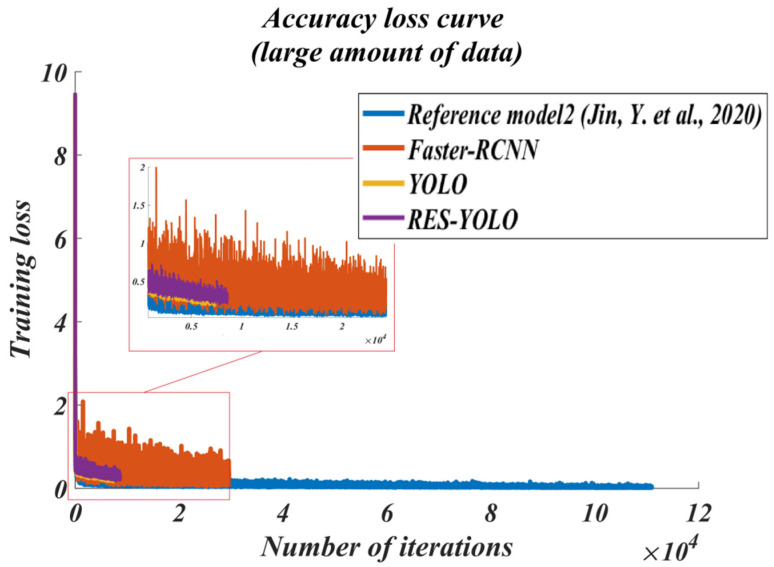
Accuracy–loss curve in small data set (large amount of data) [21].

**Figure 16:**
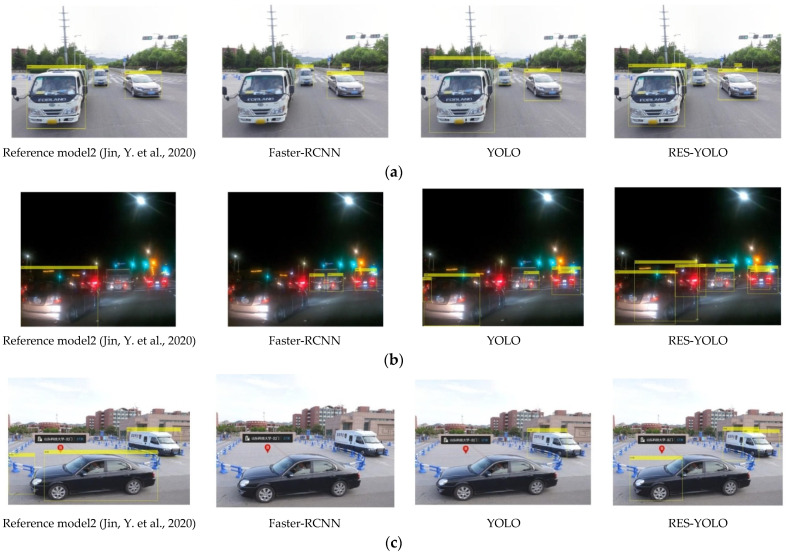
Actual test results. (**a**) Sample data 1 [21]; (**b**) Sample data 2 [21]; (**c**) Sample data 3 [21].

**Figure 17:**
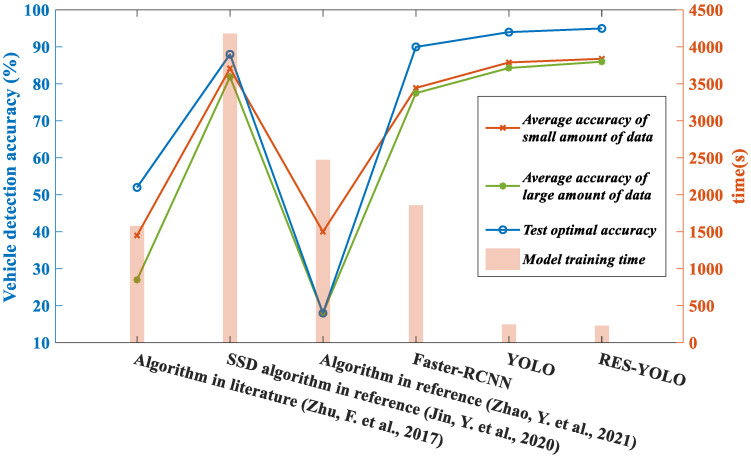
Actual test renderings of six types of networks [20,21,22].

In order to evaluate the performance of the improved RES-YOLO algorithm, the latest dual-detection framework Faster-RCNN and the single detection framework SSD are selected. The algorithms in the new references [20,21,22] are also referenced for comparison. For the convenience of description, the methods of references [20,21,22] are introduced into comparison in Figure 12, Figure 13, Figure 14, Figure 15, Figure 16 and Figure 17 of this paper. The method in reference [20] is named reference model 1, the method in reference [21] is called reference model 2, and the method in reference [22] is named reference model 3. This paper will mainly evaluate the improved YOLO algorithm from four aspects: accuracy and recall (PR) curve, accuracy–loss curve, average accuracy of test set detection, and actual detection result. When dividing the model data set, 60% of the data are selected for training, 10% are selected for verification, and 30% are used to test the trained model. The effect curves of the final test are shown in Figure 12, Figure 13, Figure 14 and Figure 15.

Through the experimental test of a small amount of data, from the accuracy–recall curves of the models, the detection effect of the models in the literature [20,22] on the vehicle targets is just average. They cannot provide a detection accuracy greater than 50% at a recall rate smaller than 0.5. The model in the literature [21] and the Faster-RCNN model can maintain a detection accuracy of 60~80% at a recall rate smaller than 0.8. The detection effects of the YOLO and RES-YOLO models are the best. Both maintain a detection accuracy greater than 90% at a recall rate smaller than 0.8. The detection accuracy of the RES-YOLO model is also 2% higher than that of the traditional models, as shown in Figure 12. From the accuracy–loss curves of the models, the overall accuracy–loss shows a downward trend, indicating that all models can complete the vehicle detection task to a certain extent. However, generally speaking, the loss curves of the models in the literature [20,22], and the Faster-RCNN models fluctuate considerably, indicating that the detection performance stability is just average, while the loss curves of the model in the literature [21] and the RES-YOLO models fluctuate less strongly, indicating that they are more suitable for vehicle detection, as shown in Figure 13.

To summarize, as the models in the literature [20,22] perform poorly under the pre-test of a small amount of data, they are far less effective than our algorithm. Therefore, they are not used for subsequent large data tests. The effects of the other models need to be further tested with the large data set group before they can be further compared with our algorithm, as shown in Figure 14 and Figure 15 below.

Through the experimental test of the large data group, from the accuracy–recall curves of models, the Faster-RCNN model has the poorest performance, followed by the model in the literature [21]. Overall, the PR curve of the YOLO algorithm is more prominent, and provides a detection accuracy within a broader range. The improved RES-YOLO algorithm also provides a better accuracy than before modification. At the same recall rate, the RES-YOLO algorithm is about 5% more accurate than the traditional YOLO algorithm, as shown in Figure 14. From the accuracy–loss curves, the accuracy–loss of the Faster-RCNN models is too heavy to complete vehicle detection. The model algorithm in the document [21] also suffers from increased accuracy–loss when performing vehicle detection tasks in multiple scenes.

In contrast, the YOLO model algorithm is obviously better than these two models. The improved RES-YOLO algorithm still performs well in accuracy–loss even after modification, as shown in Figure 15. The above results also show that by optimizing the network structure and loss function in this paper, the improved RES-YOLO algorithm can theoretically outperform the traditional YOLO algorithm and the other types of optimized algorithms. Next, we are going to perform a real-scene test.

In order to show how the RES-YOLO algorithm more intuitively outperforms the other network in vehicle detection, the measured data in different scenes are randomly collected for the actual test of the algorithm. Here, four models obtained through big data training are used, and the results are shown in Figure 16.

The results show that:

(1) The RES-YOLO algorithm can better recognize multi-vehicle targets. The improved RES-YOLO loss function effectively suppresses the influence of non-target errors on target recognition, as shown in sample data 1.
(2) The RES-YOLO algorithm can accurately recognize vehicles even in a dark (harsh) environment with high robustness. Its recognition accuracy is much higher than the algorithm in the literature [21] and the Faster-RCNN algorithm, as shown in sample data 2. Optimizing the network structure of the YOLO algorithm causes the improved RES-YOLO algorithm to be better able to deal with complex environmental information.
(3) The RES-YOLO algorithm can identify vehicle position in short-range vehicle recognition tasks and intuitively see the number of vehicles. Although the accuracy of the frame mark position still needs to be improved, the recognition effect is better than the other methods, as shown in sample data 3.

The average accuracy, training time, and optimal accuracy of the models are obtained using 10% of the validation data set, as shown in Table 4.

On this basis, five types of locally measured vehicle data sets in the network are tested. The improve algorithm can provide better recognition accuracy under different interference backgrounds, suggesting that our method is more accurate and robust for vehicle target detection than the other methods.

As shown in Figure 17, the RES-YOLO algorithm is superior to the other algorithms in operation efficiency and accuracy. As a large data volume contains more complex vehicle condition data, it is normal that the average accuracy is slightly lower than in the case of a small data volume. Nevertheless, the average accuracy of the RES-YOLO algorithm for the small data volume and the large data volume is 1.0% and 1.7% higher than the original algorithm. The training time is also 7.3% shorter. The optimal accuracy is improved by 1% to 95%. Compared with the algorithms in the literature [20,22], the accuracy of our method is improved by 47.8% and 46.8% in the case of small data volume. The accuracy is improved by 4.1% and 8.5%, respectively, in the case of large data volume. Compared with the SSD algorithm in the literature [21] and the Faster-RCNN algorithm, the accuracy is improved by 4.1% and 8.5%. Overall, the improved RES-YOLO algorithm is more suitable for vehicle target detection, suggesting that our algorithm is advantageous in vehicle target detection.

### 4.5. Experimental Test of Local Measured Data

**Figure 18:**
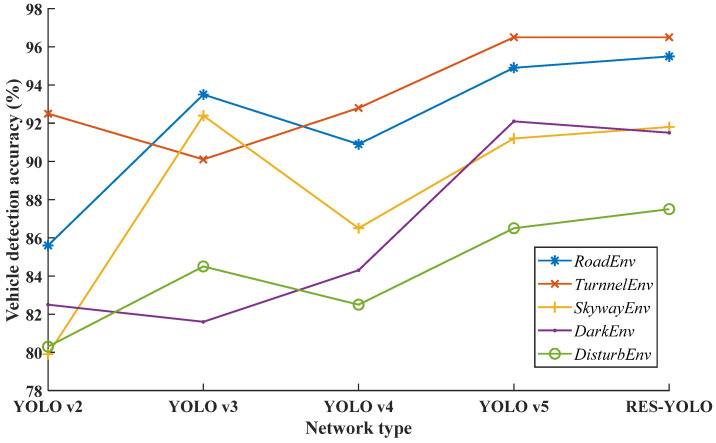
Actual test renderings of five types of networks.

**Figure 19:**
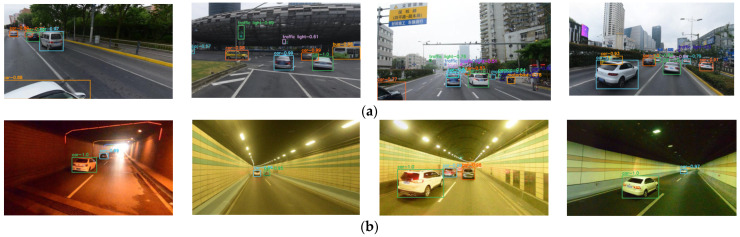

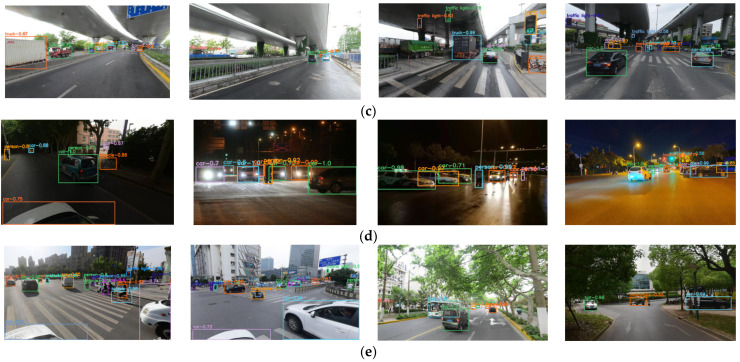
Effect diagram of actual test of RES-YOLO network in local data. (**a**) Urban road environment; (**b**) Tunnel road environment; (**c**) Skyway environment; (**d**) Night dark environment; (**e**) Trees, pedestrians, and other disturbing environments.

After the above tests, it is found that the RES-YOLO model proposed in this paper is superior to other target detection networks in the field of vehicle target detection. Nevertheless, to determine the overall performance of the YOLO v2 architecture used in this paper compared with other types of YOLO networks in vehicle detection tasks under complex backgrounds, testing of the image data measured by local researchers was performed. The vehicle conditions under different backgrounds are independently detected, and the training data set is consistent with the test in the previous section. On this basis, the vehicle detection effects of the YOLO v2 network, YOLO v3 network, YOLO v4 network, YOLO v5 network, and RES-YOLO network for the local data sets with different backgrounds are compared horizontally. The experimental results of the local test data are shown in Figure 18, and the final effect of the RES-YOLO network under the measured data is shown in Figure 19.

As shown in Figure 19, the model is further tested for six complex scenes, including: (a) urban road environment, (b) tunnel road environment, (c) skyway environment, (d) dark night environment, (e) trees, pedestrians, and other disturbing environments. Through comparative experiments, it is found that the RES-YOLO network shows the best accuracy effect in the actual tests except for the dark environment at night. As there is less background interference in a dark environment, the effect of this network is equivalent to the YOLO v5 network. In other noisy environments, the RES-YOLO network has apparent advantages. Its detection accuracy can stay above 85% in all environments, suggesting high reliability.

As shown in Figure 19, through the actual test experiment of the local data, it is found that the RES-YOLO network can perform vehicle target detection tasks in a variety of interference environments, including the urban road environment, tunnel road environment, skyway environment, night dark environment, and trees, pedestrians, and other disturbing environments. It has a satisfactory extraction effect and is well robust for vehicle target recognition in a complex background environment.

## 5. Conclusions and Discussion

Aiming at the problems encountered in the process of surveying and mapping data processing, such as difficulty in vehicle target detection, statistical errors in large data sets, and the complex automatic judgment of vehicle target self-position, the YOLO deep learning framework is studied. The traditional YOLO detection algorithm is optimized by introducing an adaptive proportional coefficient to reconstruct the loss function, and a vehicle detection model, the RES-YOLO algorithm, is proposed before it is trained and tested with the BDDK100 data set. By comparing with the latest detection methods, it is proved that the new algorithm has unique advantages in vehicle target detection. Finally, by comparing with other types of YOLO networks, it is demonstrated that the RES-YOLO network is advantageous in performing vehicle target detection tasks. The main work and conclusions of this study are as follows:

(1) The YOLO algorithm is superior to the algorithms in the literature in terms of time and accuracy. The RES-YOLO and YOLO algorithms have the highest training efficiency, followed by Faster-RCNN. The performance of the algorithms in the literature is just average.
(2) The RES-YOLO algorithm can effectively overcome background errors caused by the imbalance of positive and negative samples. It can effectively identify vehicle targets in complex backgrounds and greatly increases the usability of the YOLO algorithm.
(3) The RES-YOLO network out performs even the current mainstream YOLO-type network, especially in vehicle target detection. It can accurately identify vehicle targets for a variety of environments with good robustness.

**Figure 20:**
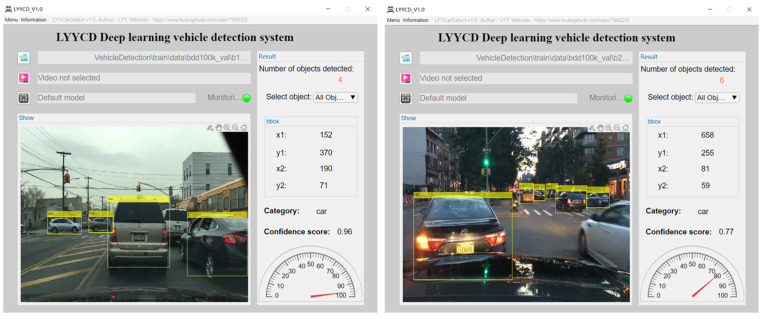
Main interface of vehicle detection system.

The research contribution of this paper is that we improved the traditional YOLO network structure, which makes it possible to recognize vehicle targets accurately and which suppresses multi-type environmental background noises effectively; moreover, we provided the optimal RES-YOLO network structure as a useful reference for the subsequent modification of the YOLO type networks for a particular target recognition. We also optimized the loss function in the YOLO network to improve its ability to suppress background noises, unitized key operations to allow for high efficiency operation, integrated our algorithm into the software application, designed an appropriate humanized interface, and incorporated a variety of picture and video detection interfaces. The main interface of the vehicle detection system, as shown in Figure 20, can be easily installed in MATLAB.

Finally, due to the diversity of conditions and data acquisition equipment, many problems with the vehicle target testing for more complex environments still need to be studied.

## Figures and Tables

**Table 1:** Data set description.

Groups	Usage	Count
Small Sample Data Group	Training Data Set	177
	Verifying Data Set	29
	Testing Data Set	89
	Total	295
Large Sample Data Group	Training Data Set	6000
	Verifying Data Set	3000
	Testing Data Set	1000
	Total	10,000

**Table 2:** Basic situation of feature extraction network.

Network Type	Network Name	Inputsize	Feature Layer Name	Complexity
Lightweight Level	AlexNet	(227 227 3)	relu5	8
	SqueezeNet	(227 227 3)	fire9-relu_squeeze1 × 1	18
	ResNet18	(224 224 3)	res5a_relu	18
	GoogleNet	(224 224 3)	pool4-3 × 3_s2	18
Robust Level	ResNet50	(224 224 3)	activation_40_relu	50
	ShuffleNet	(224 224 3)	node_126	50
	MobileNet-v2	(224 224 3)	block_13_depthwise_relu	53
	DarkNet53	(256 256 3)	leakyrelu44	53
Complex level	ResNet101	(224 224 3)	res5a_relu	101
	Inception-ResNet-v2	(299 299 3)	block8_9_ac	164
	DenseNet201	(224 224 3)	conv5_block25_1_relu	201

**Table 3:** Test of feature extraction network performance.

Category	Network Name	Average Accuracy under Small Sample Cost Test	Training Time (s)	Complexity
Lightweigh Level	AlexNet	0.59	239	8
	SqueezeNet	0.60	297	18
	ResNet18	0.67	506	18
	GoogleNet	0.59	664	18
Robust Level	ResNet50	0.86	1449	50
	ShuffleNet	0.74	436	50
	MobileNet-v2	0.71	838	53
	DarkNet53	0.85	1927	53
Complex Level	ResNet101	0.75	2684	101
	Inception-ResNet-v2	0.56	4212	164
	DenseNet201	0.54	2341	201

**Table 4:** Evaluation of multiple methods.

Method	Average Accuracy of Small Amount of Data	Average Accuracy of Large Amount of Data	Model Training Time (Small Sample)	Test Optimal Accuracy
Algorithm in literature [20]	39.0%	27.0%	1579s	52.0%
SSD algorithm in reference [21]	84.2%	81.9%	4179s	88.0%
Algorithm in reference [22]	40.0%	25.0%	2473s	18.0%
Faster-RCNN	78.9%	77.5%	1859s	90.0%
YOLO	85.8%	84.3%	247s	94.0%
RES-YOLO	86.8%	86.0%	229s	95.0%

**Figure A1:**
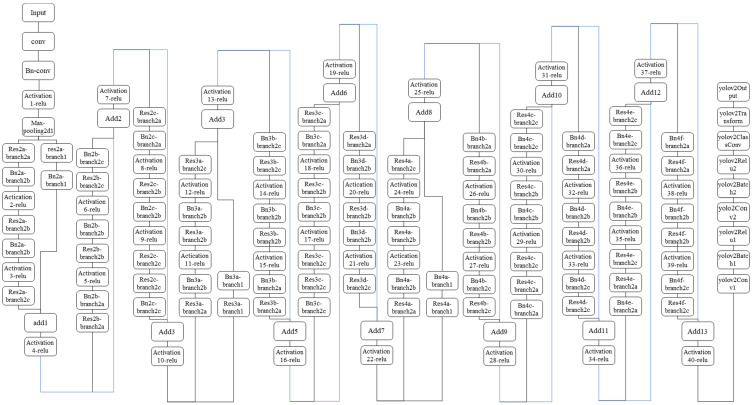
RES-YOLO Network Structure.

## Data Availability

The data used to support the findings of this study are available from the corresponding author upon request.
